# Euthyroidectomy under local versus general anesthesia in health camp settings in Uganda: a protocol for randomized prospective equivalence single-blind controlled trial

**DOI:** 10.1186/s13063-023-07387-w

**Published:** 2023-05-31

**Authors:** Umaru Kabuye, Jane Odubu Fualal, Herman Lule

**Affiliations:** 1 Department of Surgery, Kampala International University Western Campus, Ishaka, Uganda; 2 Endocrinology Unit, Department of Surgery, Mulago National Referral Hospital, Kampala, Uganda; 3 Department of Clinical Medicine, Division of Clinical Neurosciences, University of Turku, Turku University Hospital, FI-20014 Turku, Finland; 4 Department of Surgery, Kiryandongo Hospital, Kikube, Uganda

**Keywords:** Euthyroidectomy, Local anesthesia, General anesthesia, Critical care, Africa

## Abstract

**Background:**

Endemic goiter is highly prevalent in Uganda at 60.2%, contributing to the high surgical burden. While compelling evidence suggest that in selected cases, thyroidectomy under local anesthesia (LA) is associated with fewer post-operative complications, low costs, and short hospital stays, local anesthesia is not considered a priority technique for thyroidectomy in resource-constrained settings such as Uganda, despite having fewer general anesthesia (GA) and critical care providers. The objective of this trial is to compare euthyroidectomy under local versus general anesthesia among patients with grade 1–2 uncomplicated euthyroid goiter in Uganda.

**Methods:**

This prospective equivalence randomized, single-blind controlled trial protocol will be conducted among eligible participants with grade 1–2 uncomplicated euthyroid goiters. The recruitment processes will start in October 2022 and end in April 2023. Consenting participants with an indication for thyroidectomy will be randomized into two arms of 29 participants in each arm during the Bulamu Health Care Organization surgical camps in Uganda.

**Discussion:**

The primary outcome of this trial protocol is to compare the early post-operative complications of euthyroidectomy done under LA versus GA. The outcome variables include post-operative pain based on visual analogue scale, nausea, vomiting, hematoma formation, and transient voice changes determined at an interval of 6, 12, and 24 h and at 30 days.

In addition, we shall compare the surgical site infection rates, procedure costs, hospital stay, and patients’ level of satisfaction based on a 5-point Likert scale and their willingness to undergo a similar surgery using the same anesthetic technique between the two groups. We hypothesize that euthyroidectomy under LA could potentially offer similar benefits as GA, reduce costs related to procedure, complications, and hospital stay while at the same time mitigating the unmet need for surgery attributable to shortage of general anesthesia providers and critical care facilities in low-income settings.

**Trial registration:**

Pan African Clinical Trial Registry PACTR202208635457430. Registered on 11^th^ August 2022. All items from the WHO trial registration data set are within the protocol. Version number and date: version 3, 15/03/2023.

**Supplementary Information:**

The online version contains supplementary material available at 10.1186/s13063-023-07387-w.

## Introduction

Thyroid gland disorders are of global public health concern and are regarded the most common type of endocrine diseases after diabetes mellitus, accounting for 30–40% of glandular disorders [1]. Endemic goiter which is characterized by thyroid gland enlargement with or without affecting its function tops the list of thyroid gland disorders [2]. This problem disproportionately affects the poor. According to Qasim [3], there is a direct association between societal socioeconomic status, disease occurrence, and surgical outcomes that are dependent on medical care factors. As such, low-resourced countries have high prevalence of goiter, for instance up to 60.2% in Uganda [4], due to consumption of iodine-deficient meals or foods which interfere with iodine uptake. Furthermore, poorer individuals have impeded access to specialized health care and therefore present with advanced disease associated with compressive symptoms such as acute respiratory distress in addition to painful swallowing, hyperactivity of the gland (hyperthyroidism), and poor quality of life [4–6].

Thyroid surgery conventionally known as thyroidectomy is the principal treatment for symptomatic goiters that do not respond to medical treatment and remains among the most commonly performed surgical operations [6]. The operation is typically performed when the level of hormones from the gland which reflect its functionality has been normalized, hence the term euthyroidectomy.

Over time, various surgical approaches and anesthetic techniques have evolved to enable removal of a diseased thyroid gland. Historically, euthyroidectomy was associated with numerous post-operative complications that the first reported thyroid surgeon was jailed after the death of his patient in 1646 [7]. Albert Theodor Billroth was the first surgeon to popularize thyroid surgery, which encompassed primarily drainage of the thyroid cysts, although with concerns of post-operative pain and high levels of recurrence [7]. Subsequently, thyroidectomy was for years conventionally performed under regional local anesthesia (LA) resulting in loss of sensation and pain in a localized part of the body [8–10] with some series reporting favorable outcomes on over 20,000 patients as of 1990 [9, 11].

Following the advancement of general anesthesia (GA) techniques in which a state of unconsciousness is induced with complete absence of pain and reflexes, the number of thyroidectomies performed under GA increased and ultimately, GA became the preferred method under which thyroidectomy is carried out. In the current surgical practice, majority of surgeons prefer to perform thyroidectomy under GA which offers the advantage of pain-free surgery to the patient, thus reducing their anxiety and enabling a more relaxed operating room environment for the surgeon [8, 9].

Conversely, preference to GA is not without limitations. First, the longer recovery time from anesthesia results in prolonged hospitalization, delayed return to work, reduced patient turnover, and ultimately contributes to the unmet need for surgery. This culminates in patients presenting much later in life with huge goiters that have both local and systemic complications, compromising their general well-being, quality of life, and economic productivity. Secondly, in low-income settings with limited infrastructural and human resources, GA is comparatively undesirable due to concerns related to post-operative care and monitoring after the surgery such as lack of skilled staff and equipped intensive care units [6]. For instance, despite efforts from donor funding and south-west institutional collaborations, Uganda with its 45 million population boosts with only 100 qualified anesthesia providers (fewer than 0.05 anesthesia providers per 100,000 population) as opposed to high-income settings such as the UK with over 18 per 100,000 population [12]. Therefore, performing thyroidectomy under LA would offer the advantage of the anesthesia being administered by the surgeons themselves without putting much strain on the weak health systems.

Henceforth due to drawbacks of GA, in the last two decades, there has been an increase in the number of thyroidectomies performed under regional/LA combined with monitored anesthesia care [11, 13]. These numbers have even increased further with the development of minimally invasive thyroidectomy particularly in short-stay settings [9] and now, a few surgeons in the western world are currently doing thyroidectomies exclusively under LA [8].

Previous studies comparing euthyroidectomy under LA versus GA have shown mixed findings with regard to the benefits and post-operative outcomes. Other than overcoming the need to book for intensive care space and an anesthesiologist [14], which most patients in low-income countries cannot afford in the case of thyroidectomy under GA [15], LA has been associated with less post-operative pain, costs, hospital stay duration, post-operative nausea, vomiting, throat discomfort, and voice changes [9, 16, 17]. According to research in Nigeria [13], India [8], and USA [16], complications such as hematoma, surgical site infection, and colloid occur in less than 4% of cases of thyroidectomy performed under LA. In a randomized controlled study in Germany [9], the occurrences of post-operative complications (pain, nausea, vomiting, throat discomfort, painful swallowing, voice change, and difficulty in breathing were less in the group of patients who underwent thyroidectomy under LA as compared with those under GA in contrast to the prospective study in Uganda [18]. According to the USA [16] and Ugandan [18] studies, GA is also more associated with persistent hypothyroidism although the Kenyan cross-sectional review showed no difference[11].

In terms of cost, it has been arguably stated that performing thyroidectomy under LA might require additional training and capacity building in endocrine surgery which has cost implications, though comparatively similar costs would be incurred on supplies, critical care, and longer hospital stay [19, 20] more so in the error of COVID-19 where GA would increase in-patient risks. Cost of procedures is one of the limiting factors to access health care, and therefore, all measures should be taken to minimize the cost at which the surgical services are provided. A study done by Budhathoki et al. [20] in Nepal found out that the overall cost for thyroidectomy was less when done under LA as compared to when it is done under GA. The authors found that the cost of procedure included operation charge, material cost, medicines, and general ward bed charges. The mean cost in the participants that underwent thyroidectomy under GA was US $107.85 as compared to those under LA (US $65) [20]. Researchers Mamede and Raful in Brazil [19] found that the total cost of thyroidectomy was R$ 203.20 under GA compared to R$ 87.40 under LA. However, the significant difference in cost was only noticed in cost of treatment (drugs) with no significant statistical difference in the cost of hospital daily fees. In an Indian study by Shukla et al. [21], the mean cost incurred on patient from admission till discharge was Rs. 2189.32 for thyroidectomy under LA group versus Rs. 5520.00 in GA group. Such cost-effective studies are necessary in East Africa particularly in Uganda where there are budgetary constraints attached to health care, limited insurance coverage with most of the cost being paid out of pocket.

Further, in terms of patients’ level of satisfaction, which is a key patient-centered outcome, existing research in USA [16], Brazil [19], and Nepal [20] have shown no significant statistical difference in patients satisfaction for thyroidectomy done under LA versus GA and their participants would recommend to others to use either of the methods. In fact, a recent prospective comparative study in Nepal showed that the mean post-operative pain score using Likert scale was actually higher in LA versus GA group [20]. However, in East Africa where goiter is endemic, there have been no robust studies to compare the two anesthetic techniques in a randomized control setting. A Kenyan showed no difference between LA versus GA in terms of duration of surgery, operative complications, or length of facility stay [11]. Notably, the study had a smaller sample size of only 7 participants whose effect size could have limited the difference from being observed given the low incidence of such complications.

### Study rationale

According to Hisham and Aina [22], the prejudice to what technique works for the system resources is constrained by fear, doubt, and disbelief in the context of diverse cultures as to what constitutes safety, cost containment, and patient satisfaction. In addition, high level of evidence is pre-requisite to clinical application of research findings [23]. Recently, Schmidtke et al. have documented how lack of validation trials and complementary studies contribute to a huge gap between surgical research and clinical practice [23]. In this regard, until 19 March 2023, to the best of the authors’ knowledge, there are no systematic reviews and meta-analyses conducted to consolidate findings on this subject. Moreover, a majority of studies on the subject are observational and a paucity of randomized trials on the topic such as Snyder et al. [16], surgery was performed by a single surgeon with inter-operator reliability limiting the level of evidence to influence uptake into surgical practice. Validation randomized control studies are necessary to guide local medical practice particularly in low-income settings where there are competing priorities for resource allocation. To allow for local and regional comparisons, this study could inform the feasibility of using LA as a comparable alternative in the context of limited human and infrastructural resources in low-income Uganda. Most importantly, the findings will inform the applicability of either technique in surgical camp setting which is the most used avenue to address the unmet need for surgery in developing countries.

### Main objective

The aim of this randomized control trial is to compare euthyroidectomy under LA versus GA among patients with grade 1–2 goiter in Uganda.

### Specific objectives

The specific objectives of this study are as follows:

1. To compare the occurrence rates of early 30-day post-operative complications (pain, hematoma, surgical site infection) following euthyroidectomy under LA versus GA
2. To compare the cost implications of euthyroidectomy under LA versus GA
3. To compare the patients’ level of satisfaction for euthyroidectomy under LA versus GA

#### *Hypothesis*

There is no difference in the early post-operative outcomes, cost, and patients’ satisfaction between euthyroidectomy performed under LA versus GA.

### Trial design

This study will be a prospective equivalence randomized single-blind controlled trial where the outcome data collectors will be blinded about the participants’ arms. The study is designed to be implemented in accordance with the SPIRIT guidelines for clinical trial protocols [24].

### Methods: participants, interventions, and outcomes

#### Study site and settings

**Fig. 1:**
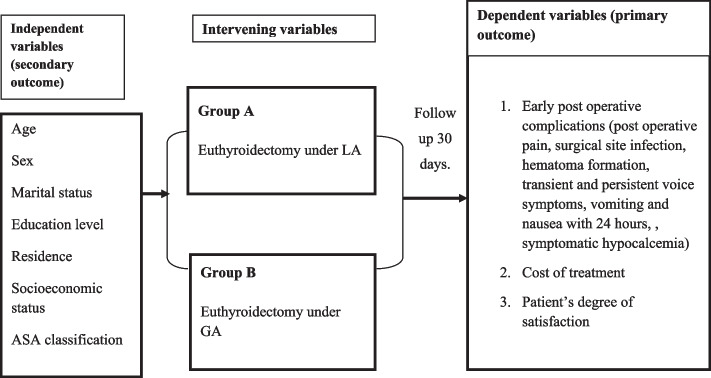
Conceptual framework (outcomes)

**Fig. 2:**
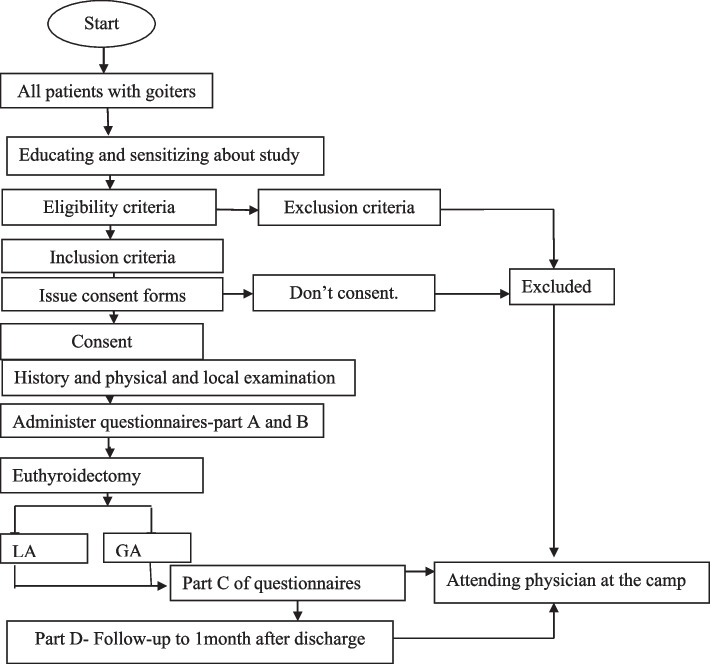
Participant timeline/study period

The study will be conducted in camp settings of Bulamu Health Care Organization that are performed monthly in different regions of the country (Uganda).The Bulamu Healthcare is a well-established non-government (NGO) not-for-profit organization that was formed in 2016 as a branch of Bulamu Healthcare International. The Bulamu Super camp model is a uniquely cost-effective program that provides health care to impoverished families of rural Uganda. Bulamu plays the role of “Great Organizer” and brings the different government agencies and non-profit NGOs together in these camps and provide free services to Ugandans. Bulamu provides medical, surgical, obstetric, and gynecological care, public health, ophthalmology, dental care, and imaging services. The camp typically attracts over 15,000 patients in a week, approximately 3000 per day. In 2019, a total of 1433 surgeries were performed of which 53 (4%) were thyroidectomies. Over 150 to 400 surgeries are performed per week. Through the third quarter of 2019, the organization managed 49,449 patients. Both LA and GA resources are available in these camps. All operations are performed by senior consultant surgeons assisted by surgery residents. The recruitment shall be done within 4–5 camps (Figs. 1 and 2).

#### Study period

The study will be limited to a period from October 2022 to April 2023; thus, May 2023 will be the follow-up month for the last recruits.

### Eligibility criteria

#### Inclusion criteria of participants

Patients between 18 and 65 years with euthyroid goiter/or made euthyroid that will attend Bulamu Healthcare camps during the study period will be given equal chance to consent and participate in the study.

#### Exclusion criteria of participants

All patients with factors that have been previously documented to independently impact the outcome of thyroidectomy and are known indications for GA [13], including:

1. Grade 3 goiter,
2. Retrosternal goiter,
3. Goiters with features of infiltration to surrounding tissues such as fixity underlying structures,
4. Obesity,
5. Previous neck surgeries, and
6. Diabetes mellitus.

### Interventions: description/study procedure

Eligible participants based on our screening log will sign an informed consent document and undergo pre-anesthetic assessment 24 h prior surgery. This assessment will be undertaken in the designated tents used for our camp settings. All participants will be subjected to the Bulamu Healthcare fasting protocol of 6 and 2 h for solid and liquid diet respectively and will receive 1 g of intravenous ceftriaxone 60 min prior surgery. It is also a routine practice in Bulamu Healthcare to record all patients’ vital signs at 24 and 0 h prior surgery. These include but not limited to pulse rate, oxygen circulation, blood pressure, and ECG monitoring. All procedures are carried out under aseptic techniques. Following the procedure, all participants will receive intramuscular injection of diclofenac sodium 7 mg stat followed by 100 mg of oral diclofenac sodium 8 hourly for 5 days. Patients will be discharged home when they score at least 9 post anesthesia discharge scoring system [25] at the discretion of attending clinicians. Review at the 30th day will be in outpatient setting upon call by the outcome assessors. Telephone follow-up interview assessment option will be available for those who will reasonably be unable to attend an outpatient visit.

### Intervention

For group L (intervention group), as adopted from a study done by Aliyu et al. [13], infiltrative local anesthesia to achieve an anterior field block in this study will involve mixing of 0.5% bupivacaine, 2% lignocaine with 1:200,000 adrenaline.

The maximum dose of bupivacaine will be 3 mg/kg and the maximum dose of 2% lignocaine with 1:200,000 adrenaline will be 7 mg/kg [13].

The total calculated dose will be made up to 60 ml using water for injection or 0.9% normal saline; 20 ml of the mixture will be used for anterior field block which extends from the supra-sternal notch below to the cricoid cartilage above to the lateral borders of sternomastoid muscles.

Ten milliliters will be given along the incision line and another 10 ml under the investing layer of the pre tracheal fascia after raising sub-platysma muscle.

The remaining 20 ml will be given at 5 ml at each of the thyroid poles to minimize discomfort during mobilization.

Patients’ position and operative field preparation will be as in surgery under GA, but during draping, patient’s mouth and nose will remain exposed to allow continued communication and spontaneous breathing.

The communication will assure the surgeon of the safety of the recurrent laryngeal nerves.

The remaining operative steps will be carried out in accordance to the standard operative texts for thyroidectomy under GA [8, 13, 17]. In brief, the steps will include (a) positioning, painting, draping, (b) skin crease-incision and sub-platysma flap, (c) incision of investing layer of deep cervical fascia, retraction of strap muscles, and or division of middle thyroid vein, (d) superior pedicle handling, (e) recurrent laryngeal nerve and parathyroid gland safeguard, (f) thyroid gland identification and inferior thyroid vessel handling, (g) thyroid bed dissection, (h) repeat steps (f-g) on the contralateral side, (i) hemostasis with or without drain placement, and (j) wound closure.

For group G (control group), euthyroidectomy under GA will be as per the routine protocol described by Aliyu et al. [13]. Not to restrict a list of drugs that might be required in emergency situations during general anesthesia, the list is left open, and costs associated will be documented for each participant.

### Interventions: modifications

#### Adverse events and trial discontinuation for individual participants

The participants are anticipated to experience pain discomfort and or other complications due to the surgery as the main treatment for goiter not because of the study. In addition to breach of trial protocols, too much pain during surgery using LA will result in converting to GA (cross over) and advancing to stronger analgesics other than prescribed in protocol with ultimate discontinuation of individual participant from the trial.

#### Interventions: adherence to intervention protocol

Inclusion and exclusion criteria will be strictly adhered to. The questionnaires shall be checked for completeness and accuracy of the data. The research assistants (doctors on duty) will be adequately trained and routinely supervised by the principal investigator and study supervisors to ensure correct use of data collection tools and adherence to ethical principles.

#### Interventions: concomitant care

No relevant concomitant care or interventions will be prohibited during the study.

#### Outcomes measures

The following outcome measures were selected based on existing literature and will be reported in conformity with Zarin et al. [26].

### Primary outcome measures

The primary outcome measures will be early 30-day post-operative complications (harms), including: pain, nausea, vomiting, hematoma, transient voice changes, and surgical site infection rates.

The complications will be reported verbally to nursing or to attending physicians for evaluation and recorded on a questionnaire in accordance with MedDRA® version 4.21 standardized reporting on medical and surgical procedures. The post-operative pain will be measured based on visual analogue scale of 0–10 where 0 means no pain and 10 means the worst possible pain. The pain will be assessed at an interval of 6, 12, and 24 h and at 30 days. The mean pain scores will be compared at using Student’s *t*-test, assuming normal distribution; otherwise, medians will be compared using non-parametric Kruskal–Wallis rank sum test.

The nausea and vomiting will be reported to nursing or attending physicians and recorded to the questionnaire using a nausea score on a scale of 0–9 where 0 means no symptom and 9 means very severe. The symptoms will be assessed at an interval of 6, 12, and 24 h and at 30 days. The mean nausea scores will be compared using a paired Student’s *t*-test, assuming normal distribution; otherwise, medians will be compared using non-parametric Kruskal–Wallis rank sum test.

Complications: hematoma, transient voice changes, and surgical site infection rates will be assessed at 6, 12, and 24 h and at 30 days will be reported in accordance with the Clavien Dindo classification of surgical complications [27], where grade I represents complications that do not require any additional intervention, grade II requires pharmacological treatment but not radiological or surgical intervention for instance blood transfusion, grade III requires radiological or surgical re-intervention, e.g., neck exploration and hematoma evacuation, grade IV representing life-threatening complications with at least one organ dysfunction such as respiratory failure that requires intensive care admission. The difference in proportions will be compared using Wilcoxon sum rank test.

### Secondary outcome measures

The average procedure costs in USD and mean duration of hospital stay (number of days away from work) will be recorded based on extracts from the patients’ case files and compared between the two anesthetic techniques.

### Tertiary outcome measures

The patients’ level of satisfaction regarding the procedure and overall experience will be reported verbally to nursing and recorded on the questionnaire based on a 5-point Likert scale where a minimum score of 1 = very dissatisfied whereas a maximum score of 5 = very satisfied. In addition, their willingness to undergo a similar surgery using the same anesthetic technique will be documented as “yes” or “no” and proportions will be compared for the two groups.

### Independent variables

Information will be captured on baseline characteristics such as sex, age, comorbidities, and ASA classification.

*Participant timeline* (see Table 1).

**Table 1:** SPIRIT timeline

	**Enrolment**	**Time of intervention**	**Post-allocation**			
Timepoint^**^	***1 week***	**0 h**	**6 h**	**12 h**	**24 h**	**30 days**
Enrolment						
Eligibility screen	X					
Informed consent	X					
Allocation	x	X				
Interventions						
Euthyroidectomy under LA or GA		**X**				
Assessments						
Socio-demographics, ASA class	X	X				
Early post operative complications			x	X	x	X
Cost implications of treatment			x	x	x	x
Patients’ level of satisfaction						X

### Sample size estimation

Sample size estimation in this study assumes a null hypothesis that there is no difference in early post-operative outcomes for euthyroidectomy under LA versus GA (equivalence study). In a study that evaluated the measurement of acute post-operative outcomes amongst patients who undergo surgery, a patient acceptable symptom state, i.e., the value beyond which patients considered themselves well, was documented at 33% [28]. Based on a study at the Scott and White Memorial Hospital, in Texas, the proportion of patients who underwent euthyroidectomy under LA with no identifiable complication were 83% as opposed to 79% who underwent GA [16]. Therefore, assuming a statistical power of 80%, type 1 error of 0.05, and a threshold for equivalence in effect with a dichotomous outcome that LA is no better or worse than GA by a minimal clinically important difference of 33% in the primary outcome measures; the sample size estimation based on Cleveland Clinic sample size estimation in clinical research (https://riskcalc.org/samplesize/) [29] will be 26 for each arm (1:1 allocation), including additional 10% to cater for loss to follow-up.

### Recruitment

This prospective equivalence randomized; single-blind controlled trial protocol will be conducted among eligible participants with grade 1–2 uncomplicated euthyroid goiters. The recruitment processes will start in October 2022 and end in April 2023.

### Methods: assignment to treatment arms

This study will be a parallel trial where different groups will be randomly allocated to receive different interventions concurrently during the study.

### Randomization, blinding and allocation concealment and implementation

Patients who meet the inclusion criteria will be assigned randomly by research assistants to either local anesthesia group (L) or general anesthesia group (G) using envelope concealment. Half of these concealed envelopes will contain letter L and the other half letter G to represent LA and GA, respectively. Permuted balanced block randomization will be performed by the study supervisor to derive a random sequence of numbers that will represent the two arms in an allocation ratio of 1:1. The random sequence numbers will be generated using a web-based software (https://www.random.org/). A set of 52 numbers will be generated beginning with a minimum of 1 and maximum of 52. The numbers will then be used to label the sealed envelopes sequentially and will be concealed to research assistants. The content of the envelope will be concealed only to be opened in the operating room for the anesthesia providers to know what anesthetic technique is to be used for the research participant.

The study participants at this point in the operating room will not be blinded as the research and ethics committee considered it a moral obligation for patients to understand the nature and risks of anesthesia techniques to be used on them prior surgery.

### Blinding

The outcome data collectors will be blinded on the anesthetic technique, thus single blinding will in effect. The biostatisticians will remain blinded until interim analysis since at this stage, the insights they would offer such when to terminate the study are presumed more important to effective delivery of the trial than the uncertain extent to which they would impart bias in the face of scarce resources [30].

### Criteria for unblinding

The outcome data collectors will only be unblinded if an adverse event related to the anesthesia intervention happens and warranty disclosure to the attending surgeons and anesthesiologists.

### Methods: data collection, management, and analysis

#### Data collection

Data will be collected by the PI and trained research assistants using a pre-designed and pre-tested structured questionnaire after educating participants for the need and significancy of follow up. The data collection tool was designed in English (the official language in Uganda) with translation in local language for the illiterate. A content validity index of 78% from 15 pre-test participants was considered adequate. Parts A and B of the questionnaire on socio-demographic and grade and size of goiter will be filled and completed. Part C of the questionnaire will be filled with intraoperative events. Part D of the questionnaire will be filled post-operatively and during follow up.

### Data collection plan: retention

A pre-designed and pre-tested structured questionnaire will be used to ensure accurate data entry. Collected data will be checked for completeness before the participant leaves the interview room, data will be collected by paper questionnaire, and then double entered in Excel 2019 version to ensure completeness and will be transferred to STATA version 15.0 for further analysis.

Participants will be continuously educated to understand the significancy of the study. The data collection tool has also been made as easy to complete as possible.

### Data management and analysis

Data will be coded, entered in Excel version 2016, and later exported to STATA 14.2. for analysis. The intention-to-treat model will be used to minimize bias as way of reflecting what happens in real clinical practice. The intention-to-treat population will be used for inference and will be defined as all eligible randomized patients recruited in this study. Analysis will be done per study objective. Socio-demographic and surgical factors will be summarized descriptively using summary measures as proportions, means, medians, and measures of variation such as standard deviations for means, interquartile range for medians. All analyses will be computed at 95% confidence intervals and regarding *p* < 0.5 as statistically significant.

## Objective one

The occurrence of early post-operative complications of euthyroidectomy under the two arms (LA and GA) of the study will be measured by total occurrence of a particular complication divided by the total number of euthyroidectomy done under LA and GA, respectively. These will be expressed as percentages with 95% confidence interval and the difference in proportions will be compared using a two-sample Student’s *t*-test, assuming normal distribution and equal variance. The Shapiro–Wilk test will be used to assess for normalcy of distribution whereas Levene’s test will be used to assess for equality of variance.

For the pain scores, visual analogue scale will be stratified as no pain (0), mild (1–3) or moderate (4–6), and severe (7–10) and the difference in proportions will be compared using non-parametric Kruskal–Wallis rank sum test (H). The overall occurrence of adverse events in both groups will be summarized as proportions in accordance with Clavien Dindo classification [27].

## Objective two

The cost of euthyroidectomy done under LA and GA will be estimated by the would-be expenditure on drugs and sundries based on national drug authority (NDA) quotation. The admission costs will be calculated in form of number days of admission (away from duty/work as cost) and bed occupancy for the two arms.

The average cost incurred on patients operated either under the LA or GA from admission till discharged will be calculated and compared. The difference in the average cost between the two arms will be compared using *t*-test, assuming normal distribution.

## Objective three

Patient’s degree of satisfaction of euthyroidectomy done under the two arms will be measured by satisfaction scale of 1/2/3/4/5 where 1 = very dissatisfied, 2 = dissatisfied, 3 = neutral, 4 = satisfied, and 5 = very satisfied.

The satisfaction will also be assessed basing on willingness to undergo a new operation (euthyroidectomy) under the same type of anesthesia. Since the satisfaction score is a Likert ordinal scale, the proportions for satisfaction scores will be computed for both arms (LA and GA) and the difference in proportions will be compared using the non-parametric test of Kruskal–Wallis (H).

### Loss to follow-up

To minimize loss to follow-up, participants will be given contacts of the study nurse coordinator who will also play a role in making reminder phone calls of those who are due for review (appointment). For those unable to come to hospital, one planned home visit will be conducted. A participant will be regarded as lost to follow-up if he or she is untraceable by hospital clinic, phone call, and home visit.

### Missing data

Values will be imputed for participants with missing end points such as withdrawals, lost to follow-up, and crossovers. The baseline clinical and sociodemographic characteristics of participants in this category will be compared between the two groups.

### Data monitoring committee and auditing for harms

#### Interim analysis

The Kampala International University Western Campus Research and Ethics Committee will form an interim committee that will audit this study and have access to results of interim analysis which will be performed when the 30th patient is recruited (58% of total sample size). The committee will independently recommend continuation, modification, or termination of the study in accordance with its ethical guidelines in relation to safety of human research participants.

Such circumstances warranting termination include unbearable adverse effects or in the unlikely event of achieving differences between study groups. Otherwise, comprehensive results would be published in the final stages of dissemination.

### Adverse events

The participants are anticipated to experience pain discomfort such as during local infiltration and or other complications due to the surgery as the main treatment for goiter not because of the study. Any unusual adverse event such as drug reaction presumed to arise from the intervention will be reported to the data monitoring committee, research, and ethics committee of Kampala International University and to the Uganda National Drug Authority.

### Individual discontinuation

Other than breach of trial protocols due to non-adherence, too much pain during surgery using LA will result in converting to GA (cross over) and advancing to stronger analgesics in the post-operative period other than prescribed in protocol. This will result in ultimate discontinuation of individual participant from the trial.

### Post-trial care

Bulamu Healthcare organization will continue with their routine follow-up of the patients and provide necessary care beyond this trial.

#### Ethics and dissemination

### Ethical clearance/approval

This trial was approved by the Kampala International University Research and Ethics committee whose official consent form document will be adopted (Ref: KIU-2022-190). The consent forms for surgery at Bulamu Healthcare organization conforms to WHO standards. All participants will have equal chance to participate as per the inclusion criteria and endorsing the consent form document will be pre-requited to participation. Participants will be able to withdraw from the study any time without compromising their entitlements.

### Protocol amendments

Any protocol amendments will be communicated to KIU-REC, trial participants, and trial registries within 24 h.

### Obtaining consent

Informed consents from participants will be obtained after full explanation of the details of the study. The details will be explained in English and or local languages (where necessary) by a well-trained study nurse from a designated tent. Signing an informed consent will constitute entry into the study (Additional file 1).

### Ancillary studies

In case of any ancillary studies, additional ethical approval and consent will be obtained from the research and ethics committee of Kampala International University before commencement of these studies.

### Maintenance of confidentiality

All study-related information will be stored securely at the study sites. Participants will be interviewed individually in private rooms. Details of research participants will be kept under lock and key for privacy and confidentiality purposes throughout the study and computer entered data will be password guarded. All data will be de-identified before dissemination.

### Dissemination policy: trial results

The results from this study will be shared with the Kampala International University Library, Director Bulamu Healthcare Organization. Findings will be published in a peer-reviewed journal.

### Dissemination policy: authorship

Individuals with substantive contributions to the design, conduct, interpretation, and reporting of this clinical trial will be granted authorship on the final trial reports in accordance with the ICMJE criteria for authorship. Author UK will take first authorship on one paper whereas author HL will take the first authorship on the second paper.

### Dissemination policy: reproducible research

Results for this study will be publicly available through open access publishing in a peer-reviewed journal. The data will be made available through a permanent weblink that will be provided by the journal for the associated data sets.

## Discussion

This trial protocol is to compare the early post-operative outcomes of euthyroidectomy done under LA versus GA. Euthyroidectomy under LA could potentially offer similar outcome as GA. Furthermore, LA could reduce costs related to procedure, complications, and hospital stay while at the same time mitigating the unmet need for surgery attributable to shortage of general anesthesia providers and critical care facilities in low-income settings.

## Study strengths and limitations

This is a randomized controlled trial expected to produce level 1 evidence. We hope to produce results that will be benchmarked for computing the statistical power for subsequent similar local protocols. Like any longitudinal study, we anticipate loss to follow-up and/or withdraw from the study, but these have been catered for in the sample size calculation.

## Supplementary Information

**Additional file 1.**

## Data Availability

Materials will be available to any scientist wishing to use them for non-commercial purposes without breaching participant confidentiality. All data will be made immediately available after publication.

## Abbreviations

KIUTH: Kampala International University Teaching Hospital
MoH: Ministry of Health
PI: Principal investigator
UDHS: Uganda Demographic Health Survey
UCG: Uganda Clinical Guidelines
WHO: World Health Organization
LA: Local anesthesia
GA: General anesthesia
NGO: Non-government organization
UMA: Uganda Medical Association
ASOU: Association of Surgeons of Uganda
NDA: National Drug Authority
