# Supplementary material for: Euthyroidectomy under local versus general anesthesia in health camp settings in Uganda: a protocol for randomized prospective equivalence single-blind controlled trial
Source: Trials. 2023 May 31;24:368. doi: 10.1186/s13063-023-07387-w (PMC10230134; doi:10.1186/s13063-023-07387-w)
Supplement: Supplementary file 1 — Additional file 1. [file 13063_2023_7387_MOESM1_ESM.docx]

**Additional files**

## Appendix I: Informed consent

**
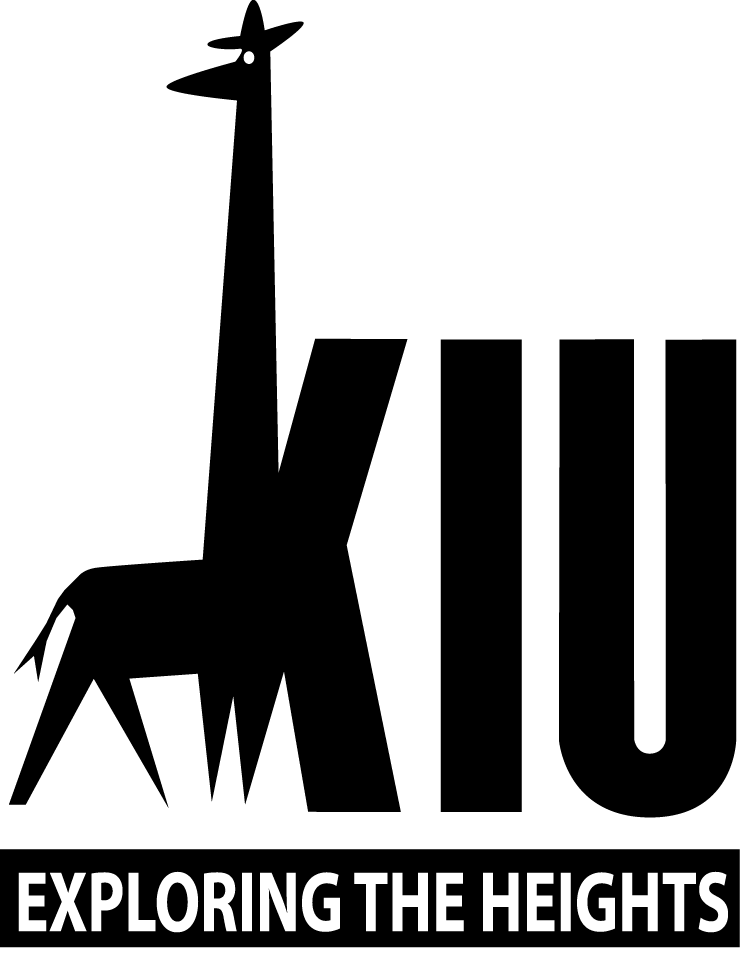
**

**Kampala International University (WIU) Western Campus (WC)**

**Research Ethics Committee (REC)**

Po Box 71, Bushenyi, Uganda; Tel:+256 758 096 775

E-mail :[kiurec2017@kiu.ac.ug](mailto:kiurec2017@kiu.ac.ug); [www.kiu.ac.ug](http://www.kiu.ac.ug)

**Informed Consent Document**

**Study title:** Euthyroidectomy Under Local Versus General Anesthesia in Health Camp Settings in Uganda: A Protocol for Randomized Prospective Equivalence Single-Blind Controlled Trial

**Principal investigator(s):** Dr Kabuye Umaru, a post-graduate student in the department of surgery at Kampala International University Teaching Hospital (KIU-TH).

**Introduction: What you should know about this study**

- You are being asked to join research study.
- This consent form explains the research study and your part in the study.
- Please read it carefully; and take as much time as you need.
- You are a volunteer. You can choose not to take part; and if you join, you may quit at any time. There will be no penalty if you decide to leave the study.

| **Leave blank (for REC office only):**  NAME OF REC CHAIR: ***Dr. Patrick Mbyemeire***  TELEPHONE: ***+256 772601482***  KIU REC STAMP: | **For REC office use only:**  APPROVAL DATE:  APPROVED CONSENT REC VERSION NUMBER:  PI’s NAME:  REC NO:***UG-REC- 023*** |
| --- | --- |

**Version: one**

**Brief background to the study**

Thyroid disorders are disease states that affect part of the body located in the front of the neck called thyroid. It is global health problem and the most common type of endocrine disorders next to diabetes accounting for 30%-40% burden of the endocrine disorder.

Thyroid diseases in sub-Saharan Africa mainly present as multinodular neck swelling called goiter. The prevalence of goiter due to iodine deficiency in the body (endemic goiter) in Uganda is 60.2%.

Thyroidectomy, the operation done to remove part of the thyroid is recently done under general anesthesia; however, it is very costly in terms of need to book intensive care unit space and medical supplies.

This results in many patients to fail to have surgery done in time when swelling is still small especially in resource limited countries like Uganda with few medical doctors trained in the field of anesthesia (anesthesiologists) and limited infrastructure.

Research shows it is feasible to do thyroidectomy by removing pain only at point of surgery (local anesthesia) with reduced costs, hospital stay duration and with good patient satisfaction. Therefore, this research will comparatively profile the operation outcomes; patient’s satisfaction and cost of euthyroidectomy under local versus general in Uganda to act as a basis for informatively consider La as an alternative mode of anesthesia during thyroidectomy in Uganda.

Therefore, as a volunteering participant you shall be asked questions along these lines of interest of this given study. And upon consenting to be part of the participants, then you shall be followed for up to 30 days after the operation.

**Purpose of the research project**

This study is intended to compare the operative outcomes, patients’ degree of satisfaction and costs of euthyroidectomy done under local versus general anesthesia in Uganda.

**Why you are being asked to participate.**

You have been selected randomly for this study because you fulfill the inclusion criteria of recruitment into this study project you are aged 18 to 65 years with a goiter that necessitates a surgical removal to be carried-out. This means that you are eligible for the study.

| **Leave blank (for REC office only):**  NAME OF REC CHAIR: ***Dr. Patrick Mbyemeire***  TELEPHONE: ***+256 772601482***  KIU REC STAMP: | **For REC office use only:**  APPROVAL DATE:  APPROVED CONSENT REC VERSION NUMBER:  PI’s NAME:  REC NO:***UG-REC- 023*** |
| --- | --- |

**Version: one**

**Procedures**

Once you meet requirements to take part in this study, you will be explained to the benefits and risks of study in detail.

Once you agree to participate in the study, you will be assigned a unique number and you shall be asked to append your signature in agreement. You will be asked a few questions about you and physical exam, and that information shall be entered in a data collecting tool and it shall be kept confidential.

Before the surgical procedure shall be undertaken, imaging and laboratory investigations may be carried-out and you shall as well sign to consent for the procedure.

**Risks / discomforts**

There are no major foreseeable risks to the participants except the risk of mild pain at point and site of drug administration (infiltration) for those that will undergo operation under local anesthesia and social harm if there is breach of confidentiality. However, social harm will be mitigated by ensuring that the most confidentiality is upheld, and risk of pain will be minimal since the procedure will be done by experienced surgeons and principal investigator who is a doctor and a master’s student of general surgery.

**Benefits**

There will be no material benefits for participating in the study. However, it is anticipated that the results of this study will guide stakeholders to develop guidelines of anesthesia for thyroidectomy to reduce on the disease burden.

**Incentives/ rewards for participating.**

There will be no cash rewards or any other incentives for participating in this study although some refreshments may be given when warranted. Only indirect benefit in form of treatment to the participants will be given.

**Protecting data management**

Privacy during interviewing and confidentiality of information are guaranteed. You will be interviewed separately from other clients and in case you know one of the researchers, you can be interviewed by someone else or withdraw from the study. You are not required to give your name to avoid information being traced back to you. The information collected will only be accessible to the research team.

| **Leave blank (for REC office only):**  NAME OF REC CHAIR: ***Dr. Patrick Mbyemeire***  TELEPHONE: ***+256 772601482***  KIU REC STAMP: | **For REC office use only:**  APPROVAL DATE:  APPROVED CONSENT REC VERSION NUMBER:  PI’s NAME:  REC NO:***UG-REC- 023*** |
| --- | --- |

**Version: one**

**Protecting subject privacy during data collection**

Data shall be obtained from private tents used during the campus. One participant shall be seen at a time.

**Right to refuse to participate or withdraw from the study.**

Participation in this study will not in any way be compulsory. Detailed information about the study will be explained to the participants and after demonstrating understanding of all the details of the study, informed consent forms will be issued and signed by the participants before the interviews and clinical examination. Any participant who will feel uncomfortable to continue with the study will be allowed to withdraw without any penalty.

**What happens if you leave the study?**

Please note that since your participation is voluntary, you have the right to refuse or may withdraw from the study at any time without compromising on any of the health care services to be offered to you by the camp. You will neither be entitled to any loss of benefits nor will be penalized for existing out of the study.

**Who do I ask/call if I have questions or a problem?**

Please contact the KIU REC office for any questions/problem on the addressed indicated below:

**The KIU REC chair**

**P.o box 71, Bushenyi**

**Mobile: +256-772601482**

**Email: kiurec@kiu.ac.ug**

**Or the** principal investigator (**Dr. Kabuye Umaru**) through the following contacts telephone number; **+256 788 609 244. / +256 759 906 618**.

**What does your signature (or thumbprint/mark) on this consent form mean?** Your signature on this form means that you have:

- Been informed about this study’s purpose, procedures, and possible benefits and risks.
- Been given the chance to ask questions before you sign; and
- Voluntarily agreed to be in this study.

| **Leave blank (for REC office only):**  NAME OF REC CHAIR: ***Dr. Patrick Mbyemeire***  TELEPHONE: ***+256 772601482***  KIU REC STAMP: | **For REC office use only:**  APPROVAL DATE:  APPROVED CONSENT REC VERSION NUMBER:  PI’s NAME:  REC NO:***UG-REC- 023*** |
| --- | --- |

**Version: one**

______________________ ________________ ___________

Name of participant signature of participant thumb print date

______________________ _____________ _____________

Name of person obtaining signature thumb print date

Consent

**_______________________ ______________ _____________**

Names of witness signature of witness thumb print date

| **Leave blank (for REC office only):**  NAME OF REC CHAIR: ***Dr. Patrick Mbyemeire***  TELEPHONE: ***+256 772601482***  KIU REC STAMP: | **For REC office use only:**  APPROVAL DATE:  APPROVED CONSENT REC VERSION NUMBER:  PI’s NAME:  REC NO:***UG-REC- 023*** |
| --- | --- |

**Version: one**

## Appendix 11: Data collection tool

Kindly respond to the following questions that form part of this data collection tool.

The rest of the tool will be filled by the investigator with data the correctly correlate to your management. If you feel any discomfort, you are free to discontinue from the study.

|  | | **PART A and B** | | | | |
| --- | --- | --- | --- | --- | --- | --- |
| **A** | | **Socio-demographics** | | | | |
| 1 | | Age ……… (Years). | | 1. 2 | Sex   1. Male 2. Female | |
| 3 | | Marital status   1. Married 2. Single 3. Cohabiting 4. Other specify……………… | | 1. 4 | Education level   1. Primary 2. Secondary 3. Tertiary 4. University 5. None | |
| 4 | | Main source of family daily income  ……………………………………… | | 1. 6 | Residence  Rural Urban | |
| **B** | | **History and preoperative examination** | | | | |
| 1 | | 1. For how long have you had this neck swelling? (months) 2. Do have any difficulty in breathing? (Yes) …. (No)…… 3. Do have any difficulty/pain on swallowing? (Yes)…... (No)…… 4. Do you have any voice changes? (Yes)…. (No)…… | 1. 2 | | Who grade of goiter?   1. Grade 1A 2. Grade 1B 3. Grade 2 | |
| 3 | | Ultrasound scan lesion size………….cm | 4 | | Any Other imaging test done.  Specify………………………. | |
| 5  7 | | Complete blood count/ hemoglobin (CBC/Hgb) (Yes)…. or (No)…….    If Yes:   1. Hgb………… (g/dl) 2. Platelets……. (mcL) 3. WBCS………. (cells/l) | 6 | | Thyroid function tests (TFTS):  (Yes)……. or (No)…………  If Yes:   1. TSH……… (mIU/l) 2. T3…………(ng/dl) 3. T4…………(ng/dl) | |
|  |  | Any Other laboratory test done:  Specify………………………. | | | | |
|  | | **PART C- Intraoperative** | | | | |
| 1 | | Type of anesthesia   1. General (GA) 2. Local (LA) 3. Conversion to GA | 1. 2 | | Type of surgery   1. Hemi thyroidectomy 2. Near total thyroidectomy 3. Total thyroidectomy | |
| **3** | | Duration of procedure…………(minutes) | | | | |
| **4** | | Intra operative drugs used | | | | |
| **5** | | \| LA \| \| GA \| \| \| --- \| --- \| --- \| --- \| \| List of drugs used \| Cost \| List of drugs used. \| Cost \| \| 1. Lignocaine  2. Bupivacaine  3. Adrenaline  Others(specify) \|  \| 1. Ketamine 2. Isoflurane 3. Propofol 4. Halothane 5. Atracurium 6. Others(specify): ……………………………………. \|  \| \| Total cost \|  \| Total cost \|  \| | | | | |
|  | | **PART D--Post operative period (up to discharge)** | | | | |
| **a** | | **Postoperative complications at 6hours, 12hours, 24hours and 30days** | | | | |
|  | | 1. Nausea score1, 2,3,4,5,6,7,8,9 (most severe possible) 2. vomiting 3. Hematoma formation 4. Surgical site infection 5. Transient voice changes 6. Permanent voice symptoms 7. Symptomatic hypocalcemia | | | | |
|  |  | **Clavien Dindo severity of complication**   1. No additional pharmacological, radiological, or surgical intervention required. 2. Requires pharmacological but not radiological or surgical intervention. 3. Requires radiological or surgical re-intervention i.e., neck re-exploration 4. Life threatening complication or at least one organ failure e.g., respiratory | | | | |
|  |  | **Post operative pain score (keeping analgesia constant)** | | | | |
|  |  | **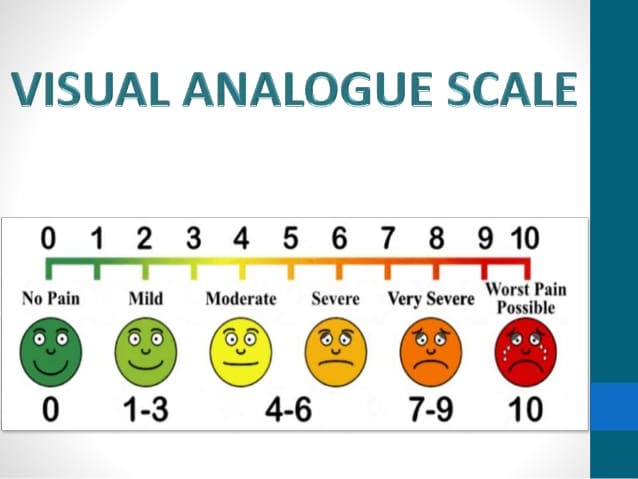** | **Time pain score**  at 6hours  at 12 hours  At 24hours | | | |
|  |  |  | **Any need for additional analgesia**  Yes……………………………  No…………………………… | | | |
|  | | Duration of facility stay after surgery.  0-<24hours 24-48hours >48hours | | | | |
| **D. Patient satisfaction with procedure at discharge** | | | | | | |
|  | Using 5-point Likert satisfaction rating scale-1/2/3/4/5 scale (tick)   \| Very dissatisfied (1)  Dissatisfied (2) \| Neutral (3)  Satisfied (4)  Very satisfied \| very satisfied (5) \| \| --- \| --- \| --- \| | | | | | Would undergo a new operation under the same type of anesthesia?  Yes No |

| SPIRIT Checklist |  | **Reporting item** | **Page and line number** | **Reason if not applicable** | |
| --- | --- | --- | --- | --- | --- |
| **Administrative information** | | | | | |
| Title | [#1](https://www.goodreports.org/reporting-checklists/spirit/info/#1) | Descriptive title identifying the study design, population, interventions, and, if applicable, trial acronym | Page1-2(upto7) | |  |
| Trial registration | [#2a](https://www.goodreports.org/reporting-checklists/spirit/info/#2a) | Trial identifier and registry name. If not yet registered, name of intended registry | Page2(line 8-9) | |  |
| Trial registration: data set | [#2b](https://www.goodreports.org/reporting-checklists/spirit/info/#2b) | All items from the world health organization trial registration data set | Page Page2(line 8-9) | |  |
| Protocol version | [#3](https://www.goodreports.org/reporting-checklists/spirit/info/#3) | Date and version identifier | Page10(line 5) | |  |
| Funding | [#4](https://www.goodreports.org/reporting-checklists/spirit/info/#4) | Sources and types of financial, material, and other support | Page 12 (line 3-4) | |  |
| Roles and responsibilities: contributors | [#5a](https://www.goodreports.org/reporting-checklists/spirit/info/#5a) | Names, affiliations, and roles of protocol contributors | Page 12(line5-10) | |  |
| Roles and responsibilities: sponsor contact information | [#5b](https://www.goodreports.org/reporting-checklists/spirit/info/#5b) | Name and contact information for the trial sponsor | N/A | | Research is fully funded by the primary investigator being academic related research |
| Roles and responsibilities: sponsor and funder | [#5c](https://www.goodreports.org/reporting-checklists/spirit/info/#5c) | Role of study sponsor and funders, if any, in study design; collection, management, analysis, and interpretation of data; writing of the report; and the decision to submit the report for publication, including whether they will have ultimate authority over any of these activities | N/A | | Research is fully funded by the primary investigator being academic related research |
| Roles and responsibilities: committees | [#5d](https://www.goodreports.org/reporting-checklists/spirit/info/#5d) | Composition, roles, and responsibilities of the coordinating center, steering committee, endpoint adjudication committee, data management team, and other individuals or groups overseeing the trial, if applicable (see item 21a for data monitoring committee) | Page 13 | | The Kampala international university research and ethics committee will form the data monitoring committee that will oversee the trial |
| **Introduction** |  |  | Page 2- | |  |
| Background and rationale | [#6a](https://www.goodreports.org/reporting-checklists/spirit/info/#6a) | Description of research question and justification for undertaking the trial, including summary of relevant studies (published and unpublished) examining benefits and harms for each intervention | Page2-4(up to line -31 | |  |
| Background and rationale: choice of comparators | [#6b](https://www.goodreports.org/reporting-checklists/spirit/info/#6b) | Explanation for choice of comparators | Page 4 (31-35) | |  |
| Objectives | [#7](https://www.goodreports.org/reporting-checklists/spirit/info/#7) | Specific objectives or hypotheses | Page5(line 4-12) | |  |
| Trial design | [#8](https://www.goodreports.org/reporting-checklists/spirit/info/#8) | Description of trial design including type of trial (e.g., parallel group, crossover, factorial, single group), allocation ratio, and framework (e.g., superiority, equivalence, non-inferiority, exploratory) | Page5 (line 14-17) | |  |
| **Methods: participants, interventions, and outcomes** | | | | | |
| Study setting | [#9](https://www.goodreports.org/reporting-checklists/spirit/info/#9) | Description of study settings (e.g., community clinic, academic hospital) and list of countries where data will be collected. Reference to where list of study sites can be obtained | Page5 (line18) –page 6(up to line18) | |  |
| Eligibility criteria | [#10](https://www.goodreports.org/reporting-checklists/spirit/info/#10) | Inclusion and exclusion criteria for participants. If applicable, eligibility criteria for study centers and individuals who will perform the interventions (e.g., surgeons, psychotherapists) | Page6(line 19-33) | |  |
| Interventions: description | [#11a](https://www.goodreports.org/reporting-checklists/spirit/info/#11a) | Interventions for each group with sufficient detail to allow replication, including how and when they will be administered | Page 8(line6-29) | |  |
| Interventions: modifications | [#11b](https://www.goodreports.org/reporting-checklists/spirit/info/#11b) | Criteria for discontinuing or modifying allocated interventions for a given trial participant (e.g., drug dose change in response to harms, participant request, or improving / worsening disease) | Page 13, 14 | |  |
| Interventions: adherence | [#11c](https://www.goodreports.org/reporting-checklists/spirit/info/#11c) | Strategies to improve adherence to intervention protocols, and any procedures for monitoring adherence (e.g., drug tablet return; laboratory tests) | Page 8-19(line31-37) | |  |
| Interventions: concomitant care | [#11d](https://www.goodreports.org/reporting-checklists/spirit/info/#11d) | Relevant concomitant care and interventions that are permitted or prohibited during the trial | N/A | | No relevant concomitant care or interventions will be prohibited during the study |
| Outcomes | [#12](https://www.goodreports.org/reporting-checklists/spirit/info/#12) | Primary, secondary, and other outcomes, including the specific measurement variable (e.g., systolic blood pressure), analysis metric (e.g., change from baseline, final value, time to event), method of aggregation (e.g., median, proportion), and time point for each outcome. Explanation of the clinical relevance of chosen efficacy and harm outcomes is strongly recommended | Page 14 | |  |
| Participant timeline | [#13](https://www.goodreports.org/reporting-checklists/spirit/info/#13) | Time schedule of enrolment, interventions (including any run-ins and washouts), assessments, and visits for participants. A schematic diagram is highly recommended (see figure) | Page 15 | |  |
| Sample size | [#14](https://www.goodreports.org/reporting-checklists/spirit/info/#14) | Estimated number of participants needed to achieve study objectives and how it was determined, including clinical and statistical assumptions supporting any sample size calculations | Page7(line 36) to page7(up to line 12) | |  |
| Recruitment | [#15](https://www.goodreports.org/reporting-checklists/spirit/info/#15) | Strategies for achieving adequate participant enrolment to reach target sample size | Page 7 line17-25 | |  |
| **Methods: assignment of interventions (for controlled trials)** | | | | | |
| Allocation: sequence generation | [#16a](https://www.goodreports.org/reporting-checklists/spirit/info/#16a) | Method of generating the allocation sequence (e.g., computer-generated random numbers), and list of any factors for stratification. To reduce predictability of a random sequence, details of any planned restriction (e.g., blocking) should be provided in a separate document that is unavailable to those who enroll participants or assign interventions | Page 7 line17-22 | |  |
| Allocation concealment mechanism | [#16b](https://www.goodreports.org/reporting-checklists/spirit/info/#16b) | Mechanism of implementing the allocation sequence (e.g., central telephone; sequentially numbered, opaque, sealed envelopes), describing any steps to conceal the sequence until interventions are assigned | Page 7-line 22-25 | |  |
| Allocation: implementation | [#16c](https://www.goodreports.org/reporting-checklists/spirit/info/#16c) | Who will generate the allocation sequence, who will enroll participants, and who will assign participants to interventions | Page 7-line 17-25 | |  |
| Blinding (masking) | [#17a](https://www.goodreports.org/reporting-checklists/spirit/info/#17a) | Who will be blinded after assignment to interventions (e.g., trial participants, care providers, outcome assessors, data analysts), and how | Page 7-line 28-29 | |  |
| Blinding (masking): emergency unblinding | [#17b](https://www.goodreports.org/reporting-checklists/spirit/info/#17b) | If blinded, circumstances under which unblinding is permissible, and procedure for revealing a participant’s allocated intervention during the trial | Page 10-line 24-26 | |  |
| **Methods: data collection, management, and analysis** | | | | | |
| Data collection plan | [#18a](https://www.goodreports.org/reporting-checklists/spirit/info/#18a) | Plans for assessment and collection of outcome, baseline, and other trial data, including any related processes to promote data quality (e.g., duplicate measurements, training of assessors) and a description of study instruments (e.g., questionnaires, laboratory tests) along with their reliability and validity, if known. Reference to where data collection forms can be found, if not in the protocol | Page 9(line 3-11) | |  |
| Data collection plan: retention | [#18b](https://www.goodreports.org/reporting-checklists/spirit/info/#18b) | Plans to promote participant retention and complete follow-up, including list of any outcome data to be collected for participants who discontinue or deviate from intervention protocols | Page 9(line 3-11) | |  |
| Data management | [#19](https://www.goodreports.org/reporting-checklists/spirit/info/#19) | Plans for data entry, coding, security, and storage, including any related processes to promote data quality (e.g., double data entry; range checks for data values). Reference to where details of data management procedures can be found, if not in the protocol | Page-9, line-12-13 | |  |
| Statistics: outcomes | [#20a](https://www.goodreports.org/reporting-checklists/spirit/info/#20a) | Statistical methods for analyzing primary and secondary outcomes. Reference to where other details of the statistical analysis plan can be found, if not in the protocol | Page 9 line (14-39) | |  |
| Statistics: additional analyses | [#20b](https://www.goodreports.org/reporting-checklists/spirit/info/#20b) | Methods for any additional analyses (e.g., subgroup and adjusted analyses) | Page 9 line (25,32,39) | |  |
| Statistics: analysis population and missing data | [#20c](https://www.goodreports.org/reporting-checklists/spirit/info/#20c) | Definition of analysis population relating to protocol non-adherence (e.g., as randomized analysis), and any statistical methods to handle missing data (e.g., multiple imputation) | Page 11, 12 | |  |
| **Methods: monitoring** | | | | | |
| Data monitoring: formal committee | [#21a](https://www.goodreports.org/reporting-checklists/spirit/info/#21a) | Composition of data monitoring committee (DMC); summary of its role and reporting structure; statement of whether it is independent from the sponsor and competing interests; and reference to where further details about its charter can be found, if not in the protocol. Alternatively, an explanation of why a DMC is not needed | Page 13 | |  |
